# Marine Algae Extract (*Grateloupia Sparsa*) for the Green Synthesis of Co_3_O_4_NPs: Antioxidant, Antibacterial, Anticancer, and Hemolytic Activities

**DOI:** 10.1155/2022/3977935

**Published:** 2022-10-20

**Authors:** Amira K. Hajri, Marzough A. Albalawi, Ifat Alsharif, Bassem Jamoussi

**Affiliations:** ^1^Department of Chemistry, Alwajh College, University of Tabuk, Tabuk, Saudi Arabia; ^2^Department of Biology, Jamoum University College, Umm Al-Qura University, Makkah 21955, Saudi Arabia; ^3^Department of Environmental Sciences, Faculty of Meteorology, Environment and Arid Land Agriculture, King Abdulaziz University, Jeddah 21589, Saudi Arabia

## Abstract

The aqueous extract of red algae was used for bio-inspired manufacturing of cobalt oxide nanoparticles (Co_3_O_4_NPs) and for antioxidant, antibacterial, hemolytic potency, and anticancer activity. Typical, characterization techniques include UV-Vis, SEM, EDAX, TEM, FTIR, XRD, and TGA. Using an X-ray diffraction assay, the size of the Co_3_O_4_NPs crystal was determined to range from 23.2 to 11.8 nm. Based on TEM and SEM pictures, biosynthesized Co_3_O_4_NPs' had a homogeneous spherical morphology with a 28.8 to 7.6 nm average diameter. Furthermore, Co_3_O_4_NPs biological properties were investigated, including determining the antibacterial potency using the zone of inhibition (ZOI) method and determining the minimal inhibitory concentration (MIC). The antibacterial activity of Co_3_O_4_NPs was higher than that of the ciprofloxacin standard. Alternatively, scavenging of DPPH free radical investigation was carried out to test the antioxidant capacitance of Co_3_O_4_NPs, revealing significant antioxidant ability. The biosynthesized Co_3_O_4_NPs have a dose-dependent effect on erythrocyte viability, indicating that this technique is harmless. Furthermore, bioinspired Co_3_O_4_NPs effectively against HepG2 cancer cells (IC_50_: 201.3 *μ*g/ml). Co_3_O_4_NPs would be a therapeutic aid due to their antioxidant, antibacterial, and anticancer properties.

## 1. Introduction

Different methods can be employed to synthesize metal oxide nanoparticles (MNPs). Each synthesis process has benefits and drawbacks. For instance, the chemical and physical approaches have several advantages, such as producing the desired size and number of nanoparticles. Still, it is eco-toxic, consumes energy, and is expensive and time-consuming [1, 2]. Biological methods include plants, algae, microbes, and other natural substances, including starch, egg albumin, and gelatin, which are used in the biological approach to produce diverse types of MNPs. This biological method is called the “green method” [3–6].

Green synthesis of MNPs is being used to solve these issues. The green-mediated technique is more advantageous than standard approaches [7–13].

Biological substances like starch and bovine albumin have also been employed in the synthesis of green MNPs [4, 14, 15].

These natural resources comprise biomolecules and metabolites that oxidize/reduce, stabilize, and produce specific MNPs with less pollution, safer, and cheaper [16].

Current advances in marine bio nanotechnology enable and drive advancements in a wide range of industries, including nanomedicine, pharmaceuticals, environmental concerns, and agriculture. Marine organisms that can survive in extreme conditions are plants, algae, bacteria, fungi, actinomyces, yeast, invertebrates, and mammals. Among the phytochemicals/metabolites they can generate are peptides, polyphenols, proteins, carbohydrate polymers, polysaccharides, sulfated polysaccharides, and polysaccharide-protein complexes such as fucoidan, carrageenan, carboxymethyl cellulose, polyglutamic acid, melanin, and others. These substances have distinct characteristics that distinguish them as pioneers in the ecologically friendly production of MNPs such as Ag, Au, Ru, Cobalt Oxide, and ZnO in a single-phase system [17].

Algae are marine microorganisms that are heavily used to synthesize MNPs. Algae are bionanofactories because they produce stable nanomaterials that do not require cell upkeep [18]. Algae contain several bioactive substances like proteins, polysaccharides, and phytochemicals with -NH_2_, -OH, and -COOH functional groups used in MNP production [19]. Algae are classified as microalgae or macroalgae [20]. The green macroalgae *Grateloupia sparsa* was used by their function group that acts as reducing and stabilizing agents to manufacture MNPs [21, 22].

Cobalt is a good transition metal for health [23]. It is a component of vitamin B12, which helps alleviate anemia by promoting the development of red blood cells [24]. Cobalt's unusual optical, magnetic, catalytic, and electrical properties make it ideal for nanosensors and nanoelectronics fields [25–27]. Cobalt is valuable in many sectors because of its CO^2+^, CO^3+^, and CO^4+^ oxidation states [28].

Dozens of studies are directed toward using metal oxide nanoparticles in many applications [12, 29–33]. The most often used metal oxide NPs are cobalt oxide (Co_3_O_4_NPs). These nanoparticles have recently gained popularity owing to their lower cost than noble metal nanoparticles. Their vast surface area gave a unique electrical and magnetic property [34]. Co_3_O_4_NPs are nontoxic at low doses, exhibit high antibacterial and antifungal activity, and have fewer adverse effects than antibiotics [34–36].

Antibiotic resistance is currently a severe global health concern. So, an antibiotic agent that can kill harmful bacteria resistant to existing antibiotics is required [37]. Because MNPs are smaller and have more surface area than larger molecules, they exhibit strong antibacterial properties. The MNPs disrupt the cell membrane and impede protein synthesis in bacteria [38]. MNPs such as cobalt oxide, iron oxide, and copper oxide all demonstrated antibacterial activity [39–41].

The Co_3_O_4_NPs may potentially be antimicrobial; the disc diffusion method was used to study the antibacterial activity of Co_3_O_4_NPs synthesized from *Celosia argentea* whole plant extract. These NPs were bactericidal against *B. subtilis and E. coli* [42]. The antibacterial efficacy of green-mediated Co_3_O_4_NPs was studied using *Hibiscus rosa-sinensis* flower extract; the results revealed a promising activity against *E. coli and S. aureus* [43]. Two main points have been raised. Co^2+^ and Co^3+^ interact with the negative charge sections of the bacteria and cause cell death. Second, light irradiation in the conduction and valence bands may excite electrons on the surface of cobalt oxide, and excited electrons and oxygen molecules react to generate a superoxide radical anion [44].

The cytotoxicity of human umbilical vein endothelial cells (HUVECs) was assessed in vitro using green-synthesized Co_3_O_4_NPs at various doses. The MTT test was performed on cells treated with varying quantities of Co_3_O_4_NPs; it revealed high viability up to 1,000 mg/mL of Co_3_O_4_NPs [45]. Also, it was found that Co_3_O_4_NPs are cytotoxic to HeLa carcinoma cells [46]. Besides, the biogenic Co_3_O_4_NPs have improved radical scavenging and reducing power [47]. According to a recent study, the scavenging capability and antioxidant properties of bio-inspired Co_3_O_4_NPs are dose-dependent [42].

Hemolysis occurs when disrupted erythrocyte membranes, cause hemoglobin leakage and possibly jaundice or anemia. The hemolytic potency of any newly synthesized pharmacological preparation must be tested [48]. Based on the hemolytic activity of green-synthesized Co_3_O_4_NPs, Shahzadi et al. results revealed that the bio-inspired Co_3_O_4_NPs had less hemolytic potency (2.95%) than the positive control triton-X-100 (95.25%) and less toxicity (1.02%) [42].

In this study, bioinspired Co_3_O_4_NPs synthesis was performed using red algae extract (*Grateloupia sparsa*) for Co_3_O_4_NPs synthesis. This metal oxide NPs characterization has been broadly done by UV, TEM, EDAX, SEM, XRD, FTIR, and TGA. Moreover, the antibacterial properties, anticancer potency, and hemolytic assay of Co_3_O_4_NPs have been studied in vitro.

## 2. Experimental

### 2.1. Chemicals

Cobalt (II) nitrate hexahydrate (Co(NO_3_)_2_.6H_2_O) for analysis (MTT, 98%, and (DPPH) were purchased from Sigma–Aldrich (USA), 99.9%Methanol, and DMEM-F12 (Merck Chemicals, Germany). Otherwise, the listed compounds are analytical grade and can be used without further purification.

### 2.2. Collection of Red Algae

The crimson algae were collected during a trip to the Red Sea. To transport the collected algae to the laboratory, they were placed in a plastic bottle. Thus, samples are washed and cleansed with running water to remove salt, toxins, and epiphytes. Then, it was dried and ground to a powder at room temperature using an electric blender.

### 2.3. Bio-Inspired Synthesis of Co_3_O_4_NPs

The algae-dried powder was utilized to prepare the extract. 5 gm of this powder was suspended in 50 mL DD water and heated the mixture to 60 C for 4 h. Then, the extracts were cooled at room temperature (R.T), they were filtered through Whatman filter paper and kept at 4 C. After that, 10 ml of algae extracts were injected dropwise with 50 ml of cobalt nitrate at a concentration of 1 mg/ml as a source of cobalt, following which, at R.T, continual stirring was performed. Within 24 hours of incubation, the solution's color changes from pink to brown, indicating the creation of Co_3_O_4_NPs.

### 2.4. Co_3_O_4_NPs Characteristics

The UV-Vis spectrophotometry (dual beam, Shimadzu, 1900, Japan) was used to determine the production of Co_3_O_4_NPs at wavelengths between 300 and 600 nm. The FTIR-6800 Spectrometer (JASCO, 500–4000 cm^−1^) was used to determine the functional moieties. All samples were subjected to X-ray diffraction (XRD, Philips X Pert diffractometer, The Netherlands) to validate the crystallinity and size of the Co_3_O_4_NPs. Additionally, the form and size distribution of the particles were investigated by scanning electron microscopy (SEM, FEI Quanta 200 FEG, Japan). Additionally, the elemental composition was identified by an EDAX study. Further morphological images of Co_3_O_4_NPs were examined using 120 kV transmission electron microscopy (TEM, JEOL, and JEM 1400).

### 2.5. Biological Properties of Co_3_O_4_NPs

#### 2.5.1. Antibacterial Potency

Antibacterial examinations against two G-negative and two G-positive bacteria (*E-coli and P. aeruginosa*) and (*S. aureus* and *B. subtilis*), respectively, were conducted in vitro using the zone of inhibition (ZOI) method by culturing the bacteria on Petri dish nutrient agar. Then, 6 mm filter discs containing 20 *μ*g/ml of Co_3_O_4_NPs were put on bacterial streaks, and discs with ciprofloxacin (30 *μ*g/ml) were frequently placed in the same dish as standard antibiotics. Finally, all Petri plates were incubated for 24 h at 37 C to compute the inhibitory zone [8, 49].

#### 2.5.2. Measurement of Minimal Inhibitory Concentration (MIC)

MIC values were measured using Sarker's broth agar dilution method [50]. 100 ml of Co_3_O_4_NPs (2 mg/ml) were placed on the plate's initial row, and 50 *μ*l of nutritional broth agar was applied to the other wells. After that, serial dilutions were conducted using sterilized pipettes in 1000 to 3.90 *μ*g. The resazurin solution was produced by mixing 260 mg in 50 *μ*l of sterile distilled water. All wells were treated with the resazurin solution (10 *μ*l). Also, 30 *μ*l of nutritional broth was completed to a total capacity of 100 *μ*l. Finally, 10 *μ*l of culture suspensions were mixed with the contents of the wells, and then, the plate was incubated for 24 h at 37 C, and the color change was photometrically determined. The color change from colorless purple to beginning purple was a desirable outcome. The lowest MIC value in, which the solution becomes colorless [51].

#### 2.5.3. Anticancer Potency

We evaluated the antitumor activity of bioinspired Co_3_O_4_NPs utilizing the hepatic cancer cell line (HepG2) using the MTT test. Streptomycin and penicillin (1%) were added to DMEM for cell development at 37 C in a 5% CO_2_ incubator. Additionally, different doses of Co_3_O_4_NPs (50–500 *μ*g/ml) were incubated for 48 h at 37 C in a 96-well plate. Then, each well was loaded with 20 *μ*l MTT solution and incubated for 3 hours. Finally, 100 *μ*l of DMSO was applied to the culture and incubated for 25 minutes; formazan production by live cells was determined using an Elisa reader set to 570 nm wavelength [52].

#### 2.5.4. Hemolytic Activity

A standard method was used to determine the hemolytic property of Co_3_O_4_NPs. 3 ml of freshly prepared K_3_-EDTA human blood was withdrawn and centrifuged for 5 minutes at 1500 rpm. Following that, the plasma was removed, and 2 ml of phosphate-buffered sterile saline (PBS) was added, followed by 5 minutes of centrifugation at 1500 rpm to remove any remaining PBS. The first human blood tube was filled with 100 *μ*l of Co_3_O_4_NPs and incubated for 35 minutes at 37 C. The tube was then placed in a cold bath for 5 minutes before centrifuging at 1500 rpm for 5 minutes. The supernatant was diluted (1 : 10) with cooled PBS (4 C) [43]. The same procedure was used in the tube with PBS and 0.1% Triton X-100 as a negative and positive control, respectively. Finally, each sample's optical density (OD) was measured using *k* 576 nm. The following equation was used to measure the proportion of erythrocyte lysis in each sample:

(1) $$\begin{array}{c} Hemolysis\left(\%\right)=\frac{sample\left(OD\right)-blank\left(0D\right)}{positive\;control\left(0D\right)}\times 100\text{.} \end{array}$$

#### 2.5.5. Antioxidant Property

Spectrophotometric techniques were used to evaluate the acceptor activity of the DPPH. To make a stock solution, 25 ml of methanol was mixed with 2.5 mg of DPPH as a free radical. Individually, different amounts of Co_3_O_4_NPs ranging from (50–500 *μ*g/ml) were added to a microplate. Then, 100 *μ*l of working solution were added to the microplate, covered, and incubated in the dark for 25 minutes. The activity of the radical scavenger was then evaluated by measuring the OD at 517 nm with a spectrophotometer [53]. All measurements were taken in triplicate.

The following equation is used to measure antioxidant properties:

(2) $$\begin{array}{c} DPPH\;Scavanging\left(\%\right)=\frac{control\left(OD\right)-sample\left(OD\right)}{control\left(OD\right)}\text{.} \end{array}$$

## 3. Results and Discussion

### 3.1. Co_3_O_4_NPs Characteristics

#### 3.1.1. UV-Vis

**Figure 1:**
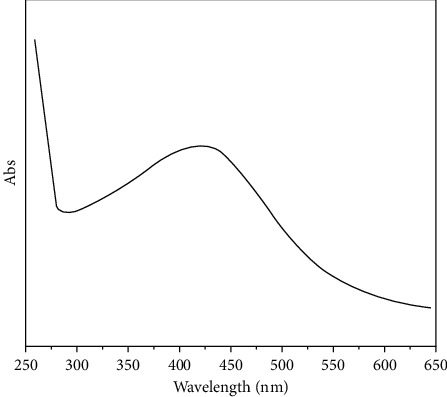
UV-vis spectra of green synthesized Co_3_O_4_NPs.

One of the main structural description methods for metallic oxide nanoparticles is UV-Vis spectroscopy.  Figure 1 depicts the UV-Vis spectra of benign Co_3_O_4_NPs synthesized by an aqueous extract of red algae. The surface plasmon resonance of Co_3_O_4_NPs is near 510 nm, lightly shifted from the broad to the long-wavelength area, confirming the Co_3_O_4_NPs formation. This tiny wavelength band was owing to transverse electrical oscillation. When Co_3_O_4_NPs particle size increased, the location and morphology of SPR were likewise shifted toward longer wavelengths [54].

#### 3.1.2. FTIR

**Figure 2:**
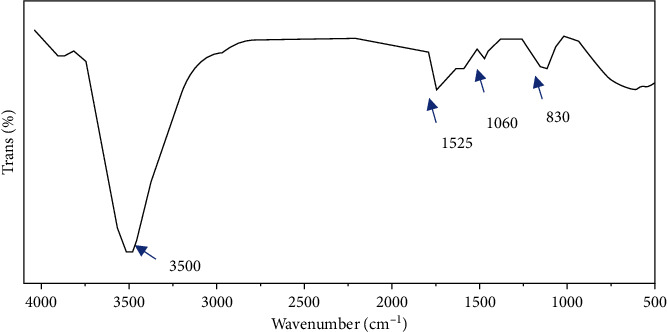
FTIR of Co_3_O_4_NPs.

Using Fourier transform infrared spectroscopy, the functional moieties of the as-prepared Co_3_O_4_NPs in Figure 2 were examined (FTIR). The band at 3500 cm^−1^ represents the -OH, whereas the bands at 1525 cm^−1^ and 1060 cm^−1^ indicate the aromatic rings and the C=O group, respectively.

#### 3.1.3. XRD

**Figure 3:**
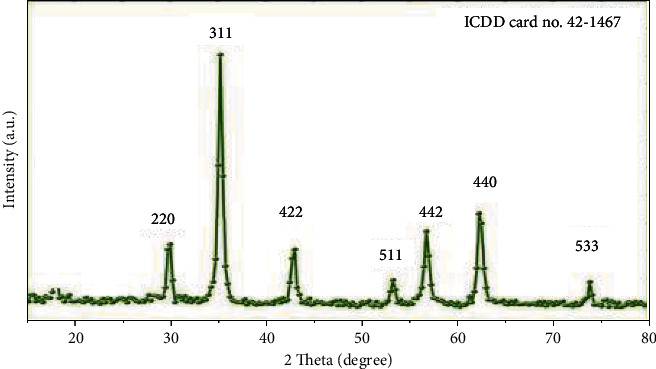
XRD of the crystalline Co_3_O_4_NPs powder.

The XRD values are depicted in Figure 3. Large diffracted intensities were recorded around 2 h = 28.2°, 35.1°, 43.6°, 53.4°, 56.3°, 63.2°, and 74.2°, which correspond to (220), (311), (422), (511), (442), and (440), respectively (533). This demonstrates the formation of the Co_3_O_4_NPs crystalline phase according to ICDD card no. 42–1467. The crystallite size is around 23.2 to 11.8 nm, according to Scherrer's equation. Low crystallinity has been associated with a greater susceptibility to lattice deformation; this could contribute to a greater affinity for chemical adhesion with the external environment.

#### 3.1.4. SEM

**Figure 4:**
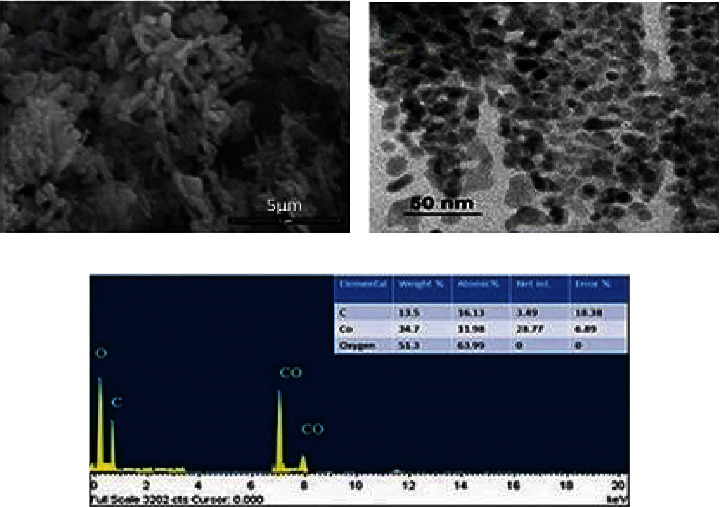
SEM micrograph of Co_3_O_4_NPs (a), TEM morphology 50 nm (b), and EDAX of bioinspired prepared Co_3_O_4_NPs (c).

SEM was used to scan the Co_3_O_4_NPs surface and size structure, demonstrating the average homogeneous formation of Co_3_O_4_NPs with a 28.2 nm diameter. SEM images of Co_3_O_4_NPs at various magnifications are shown in Figure 4(a). As explained, the nanoparticles clump together and form massive particles. The aggregation of nanoparticles has been described as an indication of metallic nanoparticle production processes [55, 56].

#### 3.1.5. TEM and EDAX

A TEM of Co_3_O_4_NPs surface morphology was displayed in (Figure 4(b)). The particle size distribution on the TEM graphic showed that the Co_3_O_4_NPs were 28.8 to 7.6 nm in size. The particles seem to be spherical as well SEM and TEM investigations produced similar findings for a wide nanoparticle size range. Based on our findings, recent studies revealed that cobalt ferrite nanoparticles made from aqueous extracts of sesame ranged from 3.0 to 20.0 nm in size [57]. *Nerium Indicum* and *Conocarpus erectus* methanol extracts were also employed to biosynthesize Co_3_O_4_NPs ranging from 20 to 60 nm in particle sizes [58]. Furthermore, the size of *Moringa oleifera* extract-biosynthesized cobalt nanoparticles ranges from 20 to 50 nm [59]. According to energy dispersion analysis, the elemental contents of materials were established by high-resolution EDAX (Figure 4(c)). EDAX of Co_3_O_4_NPs was performed in the 0 to 20 keV range. It revealed a 7 keV Co_3_O_4_NPs peak [42]. The EDAX profile had a strong cobalt signal and several short peaks [60].

#### 3.1.6. TGA

**Figure 5:**
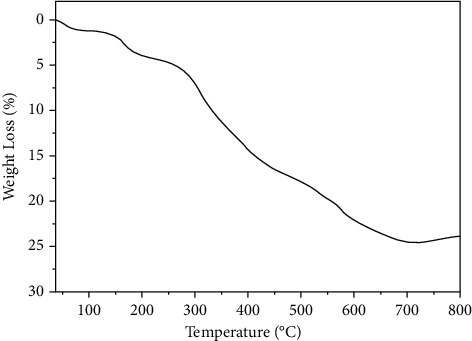
TGA of Co_3_O_4_NPs up to 800 C.

The Co_3_O_4_NPs' thermal stability is essential in evaluating their ability to survive various utilities such as fuel cells and conductor-based applications. As a result, thermogravimetric analysis was performed up to 800 C. The pristine composition declines exponentially, reaching approximately 260 C to lose about 6.3% of its original weight, which could be attributable to the release of organic solvents and water, as seen in Figure 5. A brief plateau distinguishes the second phase of weight loss between 260 and 410 C, followed by a high breakdown rate and the losses, which in this case, was ∼17.6%. Co_3_O_4_NPs appear more thermally stable than the former Co_3_O_4_NPs/algae, particularly in the first phase of thermal degradation due to the decomposition of organic species than reported in the literature [43, 61]. It is worth noting that Co_3_O_4_NPs/algae are slightly more stable below 420 C. As a result, the presence of algae may provide a good thermal scaffold for preserving stability at temperatures below 420 C.

### 3.2. Biological Properties

#### 3.2.1. Antibacterial Potency

Bacterial infection is the main serious problem in infectious illnesses in terms of death and morbidity and treatment costs [62]. Antibiotic usage has also been related to many issues, including bacterial resistance, among others. As a result, researchers strive to develop novel strategies to lower the likelihood of infectious diseases starting and spreading [32]. The rapid advancement of nanotechnology will provide tools for creating new substances with novel antibacterial properties [63, 64]. Several investigations into the antibacterial potency of biogenic metal nanoparticles have been published, with promising findings against various bacteria strains [4, 12]. At a dose of (30 *μ*g/ml), bio-inspired Co_3_O_4_NPs were tested against various bacterial species. G-positive bacteria were (*B. subtilis and S. aureus)*, whereas G-negative bacteria were (*P. aeruginosa and E. coli)* compared to the standard antibiotic ciprofloxacin disk (30 *μ*g/ml). We found that Co_3_O_4_NPs were effective on candidate bacterial species but still lower than the effect of ciprofloxacin based on ZOI measurements. On the other hand, *P. aeruginosa* with low sensitivity at a MIC of 23.0 ± 5.3 *μ*g/ml and *B. subtilis* are highly sensitive to bio-inspired Co_3_O_4_NPs, with MIC values of 18.6 ± 3.8 *μ*g/ml.  Table 1 depicts MIC values and the ZOI for antibacterial activity are compared to effective ciprofloxacin.

**Figure 6:**
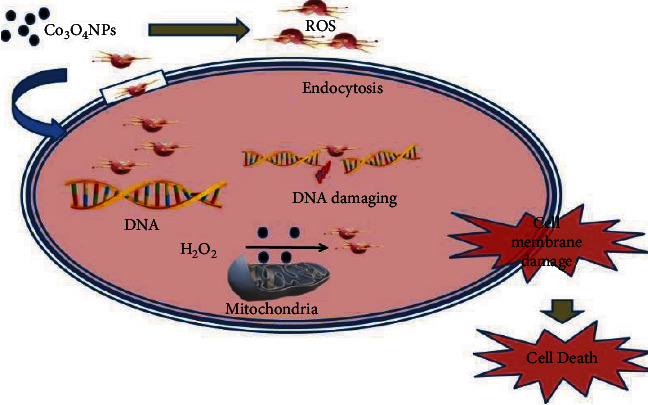
The illustration of the Co_3_O_4_NPs antibacterial mechanism.

A G-positive bacteria cell wall is composed of peptidoglycan layered with (∼70 nm thick), which permits Co_3_O_4_NPs to interface directly with the outer membrane of bacteria more readily than G-negative bacteria, which have a layer of lipopolysaccharides (1-2 mm thick) [65]. This variety in bacterial cell wall structure and thickness makes G-positive bacteria's membrane rupture faster and leads to their death [66]. As a result, the antibacterial activity of Co_3_O_4_NPs can be compact in size, and a high surface-to-volume ratio allows them to interface with the bacterial cell membrane.  Figure 6 depicts how green Co_3_O_4_NPs work against bacteria by attaching to the bacterial cell wall and modifying its permeability [67]. The penetration of reactive oxygen species (ROS) into the cytoplasm damaged the nucleus and plasmid, causing a shift in cell signaling and, eventually, death [68].

#### 3.2.2. Anticancer Potency of Co_3_O_4_NPs

**Figure 7:**
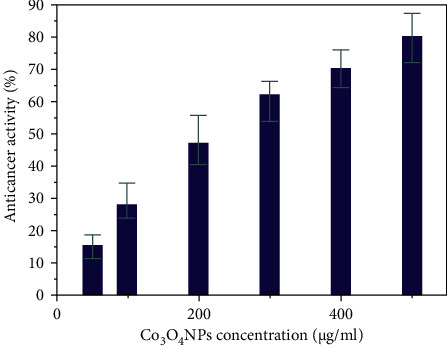
The anticancer activity of Co_3_O_4_NPs against HepG2 cell line in-vitro.

Cancer remains the world's most prominent cause of mortality. The number of cancer cases has been steadily increasing, and it is expected to reach around 21 million by 2030 [69, 70]. Hepatic cancer is the 2nd common cause of mortality in males and the 6th common cause of death in females. Excessive alcohol intake over a long period of time, as well as HCV and HBV infections, and other toxins, all increase the risk [71]. Using the HepG2 cell line, the anticancer effects of Co_3_O_4_NPs were also studied. Cancer cells were applied to various doses of Co_3_O_4_NPs (50–500 *μ*g/ml) over 24 h. In this study, Co_3_O_4_NPs were discovered to have significant anticancer potential, with an IC_50_ value of 201.3 *μ*g/ml.  Figure 7 revealed an estimated 80% fatality rate at 500 *μ*g/ml. Our findings show that Co_3_O_4_NPs generate ROS that interacts with cells and causes cellular oxidative stress, leading to DNA destruction and cell death. This is because the microscopic nanoparticles are soluble in the internal acid medium, which has a pH of 4.5. The Co_3_O_4_NPs can create pores in the membrane and dissolve in the cells, and eventually, the cell dies [72]. Cancer cells were also suppressed by Co_3_O_4_NPs, suggesting their anticancer potential. Our findings consistently show that metal nanoparticles have a significant anticancer potential [73].

#### 3.2.3. Hemolytic Potency

**Figure 8:**
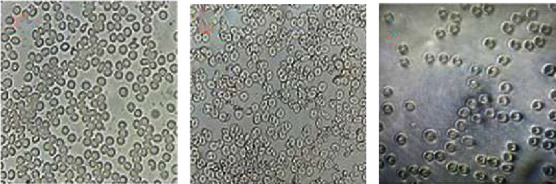
The hemolytic potency of Co_3_O_4_NPs (a), Triton-X-100 (b), and PBS (c).

The hemolytic potency of Co_3_O_4_NPs was compared to that of Triton-X-100, which represents a negative control, with Co_3_O_4_NPs made from red algae extract being significantly less toxic (5.3%) (Figure 8(a)), Triton-X-100 having 97.3% toxicity (Figure 8(b)), and PBS having 1.01% toxicity (Figure 8(c)).

#### 3.2.4. Antioxidant Activity

**Figure 9:**
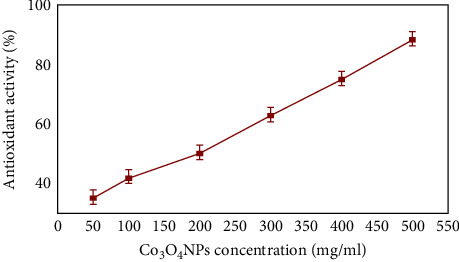
The scavenging activity of Co_3_O_4_NPs for DPPH radicals.

A DPPH-free radical scavenging test was used to evaluate the free radical scavenging capability of green-produced Co_3_O_4_NPs. These results revealed four different Co_3_O_4_NP concentrations; the free radical scavenging capability increased as the Co_3_O_4_NP concentration rose (Figure 9). The peak of DPPH radical scavenging (88.2%) was observed at 500 mg/ml. The lowest DPPH radical scavenging was observed at 50.1 mg/ml (35.0%), whereas the highest DPPH radical scavenging was obtained at 500 mg/ml (88.2%). Co_3_O_4_NPs are hypothesized to operate as electron donors, interacting with free radicals and converting them into more stable molecules capable of stopping the radical chain reaction [48].

## 4. Conclusions

Red algae were employed to create eco-friendly cobalt oxide nanoparticles (Co_3_O_4_NPs). The Co_3_O_4_NPs were formed in inhomogeneous spheres with diameters ranging from 28.8 to 7.6 nm. The antibacterial activity of Co_3_O_4_NPs was examined, and it was revealed that nanoparticle concentrations of 30 *μ*g/ml widened the inhibition zone against candidate species from 11.7 to 17.6 mm, still lower than standard antibiotics with a ZOI of 18.1 mm in its higher efficacy. Furthermore, the minimal inhibitory concentrations for (*P. aeruginosa, E. coli, S. aureus, and B. subtilis*) were adjusted to be around 23.0, 21.1, 20.6, and 18.6 for each bacterial species. Furthermore, Co_3_O_4_NPs were investigated for anticancer activity in vitro against the HepG2 cell line. Cell mortality for 500 *μ*g/ml was reported to be more than 80% after 24 hours of exposure. Furthermore, the antioxidant activity was studied, and it was observed that the maximum radical scavenging of DPPH was attained at 500 mg/ml of Co_3_O_4_NPs (88.2%).

## Figures and Tables

**Table 1:** The ZOI in petri dish agar and MIC of Co_3_O_4_NPs for bacterial growth inhibition.

		G-negative bacteria		G-positive bacteria	
		*E. coli*	*P. aeruginosa*	*B. subtilis*	*S. aureus*
*Compound*	*Dose*	Zone of inhibition (ZOI mm)			
Co_3_O_4_NPs	30 *μ*g/ml	11.7 ± 3.2	12.5 ± 3.9	14.3 ± 3.1	17.6 ± 4.2
Ciprofloxacin	30 *μ*g/ml	13.6 ± 3.4	12.7 ± 3.4	14.8 ± 2.6	18.1 ± 5.8
		Minimal inhibitory concentration (MIC)			
		MIC (*μ*g/ml)	MIC (*μ*g/ml)	MIC (*μ*g/ml)	MIC (*μ*g/ml)
Co_3_O_4_NPs		21.1 ± 6.1	23.0 ± 5.3	18.6 ± 3.8	20.6 ± 5.9

## Data Availability

The data supporting this study's results are available upon request from the corresponding author.
